# Learning from doing the EquitAble project: Content, context, process, and impact of a multi-country research project on vulnerable populations in Africa

**DOI:** 10.4102/ajod.v3i2.89

**Published:** 2014-10-06

**Authors:** Mac MacLachlan, Mutamad Amin, Gubela Mji, Hasheem Mannan, Joanne McVeigh, Eilish McAuliffe, Elina Amadhila, Alister Munthali, Arne H. Eide, A. Kudakwashe Dube

**Affiliations:** 1 Centre for Global Health and School of Psychology, Trinity College Dublin, Ireland; 2 Centre for Rehabilitation Studies, Stellenbosch University, South Africa; 3 Research & Grants, Ahfad University for Women, Omdurman, Sudan; 4 Nossal Institute for Global Health, University of Melbourne, Australia; 5 Multidisciplinary Research Centre, University of Namibia, Namibia; 6 Centre for Social Research, University of Malawi, Malawi; 7 SINTEF Health, SINTEF, Oslo, Norway; 8 Secretariat of the African Decade of Persons with Disabilities, Pretoria, South Africa

## Abstract

**Background:**

The ‘EquitAble’ project carried out content analyses of policies and collected and analysed qualitative and quantitative data concerning access to health services in Sudan, Malawi, Namibia and South Africa. Our particular concern was to address the situation of people with disabilities, although not in isolation from other marginalised or vulnerable groups.

**Objectives:**

This article reports on the content, context, process and impact of project EquitAble, funded by the European Commission Seventh Research Framework Programme, which brought together researchers from Ireland, Norway, South Africa, Namibia, Sudan and Malawi.

**Method:**

After the 4-year project ended in February 2013, all members of the consortium were asked to anonymously complete a bespoke questionnaire designed by the coordinating team. The purpose of the questionnaire was to capture the views of those who collaborated on the research project in relation to issues of content, context, process and impact of the EquitAble project.

**Results:**

Our results indicated some of the successes and challenges encountered by our consortium.

**Conclusion:**

We identified contextual and process learning points, factors often not discussed in papers, which typically focus on the reporting of the ‘content’ of results.

## Introduction

‘EquitAble’ is the acronym given to a project funded by the European Commission Seventh Framework Programme (FP7) with the full name: ‘Enabling universal and equitable access to healthcare for vulnerable people in resource poor settings in Africa’. Whilst publications from EquitAble address the situation of people with disabilities and other marginalised or vulnerable groups with regards to access and quality of health services, this article is concerned with the content, context, process and impact of the research project from the perspective of the researchers’ consortium. The aim was to learn key lessons from this comprehensive collaboration that could be utilised in future complex international research studies.

### Background

EquitAble was classified as a ‘Collaborative Project’, the proposal being submitted in response to a call in 2007 by the name of ‘HEALTH – 2007 – 3.5–2’, under the subcategory of ‘Universal and equitable access to health care and health financing’. The consortium was coordinated by Trinity College Dublin in Ireland. The other European partner was a large independent research organisation, SINTEF (*Stiftelsen for industriell og teknisk forskning*) in Norway. African partners were Ahfad University for Women in Sudan, the Centre for Social Research (CSR) at the University of Malawi, the Multidisciplinary Research Centre (MRC) at the University of Namibia, the Human Sciences Research Council (HSRC) of South Africa (which was a research agency rather than a funder), and the Secretariat of the African Decade of Persons with Disabilities (SADPD), a civil society organisation in South Africa working across African countries and partnered with the African Union, African governments, civil society organisations and disabled persons’ organisations, to promote inclusive development and human rights for people with disabilities. The Department of Psychology and Centre for Rehabilitation Studies, both at Stellenbosch University, South Africa, were also included (see EquitAble project website: http://www.equitableproject.org).

The project was carried out from March 2009 until February 2013 with a particular focus on disability. People with disabilities were amongst its researchers, and organisations representing persons with disabilities were consulted. We also undertook extensive survey sampling of people with disabilities representing different cultures and contexts across 17 sites in the four project countries.

### Research proposal development

In order to develop a comprehensive research proposal involving eight distinct institutions, the members of the consortium were required to meet in person to discuss the core elements of the proposal. Such a meeting was made possible by funding from the Health Research Board Networking Grant and Enterprise Ireland Networking Grant. The first of the two meetings was convened in Cape Town in June 2007 and the second in Dublin during July 2007. At the first meeting, participants were from Trinity College Dublin, Stellenbosch University, SADPD, and SINTEF. In the subsequent meeting all members of the consortium gathered to assist the team at Trinity College Dublin to coordinate and host a meeting of likely partners. The members worked over 3 days to develop the concept that framed the proposal submission. In addition to the meetings in Cape Town and Dublin, extensive electronic communication in relation to different versions of the proposal took place, providing the participants with further opportunities to influence the planned research study. All institutional members of the consortium had worked previously with at least one other institution in the consortium, and some with several institutions. Established research relationships strengthened communication between partners and greatly contributed to creating a genuine collaborative working relationship during the development of the proposal.

**FIGURE 1:**
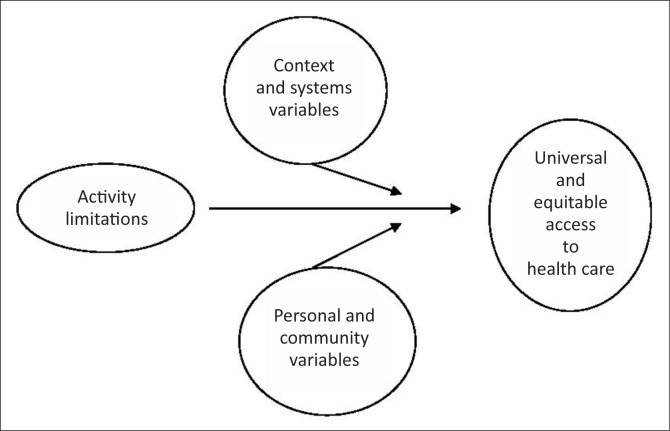
Schematic summary of the theoretical model on which EquitAble was based.

The crux of the consortium’s argument was that health care can neither be universal nor equitable if it is less accessible to some sections of society than to others. Figure 1 illustrates schematically how we operationalised this: people with disabilities are distinguished by various activity limitations (including physical, social and psychological barriers). The extent to which these barriers impede access to health care is influenced by local contextual and health systems variables, and by characteristics of the individuals and the communities in which they live.

Having identified and mutually agreed on partners who could work well together, we also sought to maximise the benefit of including the four African countries, where data collection was actually going to take place. Why these four countries? What was it about this combination of countries that added value over any other group of countries? After teasing out distinguishing contextual factors in each country we recognised that these four countries allowed us to explore access to health care systems in contexts where a large proportion of the population has been *displaced* (Sudan); where the population is highly *dispersed* (Namibia); where *chronic poverty* and high disease burden compete for meagre resources (Malawi); and where, despite *relative wealth*, universal and equitable access to healthcare is yet to be attained (South Africa) (see MacLachlan *et al*. 2012). We also sought to explore how activity limitations across the mentioned contexts interact with other factors that make people vulnerable to poor access to health care, such as age, gender, ethnicity and locality.

### Work packages

EquitAble comprised five work packages (WPs): coordination (WP1), policy analyses (WP2), a comprehensive qualitative study (WP3), extensive household surveys (WP4), and dissemination (WP5). Each of the methods chosen contributed different types of data that together yielded a more complete knowledge base (Brannen 2005:182). Data collection was carried out in all countries and, in principle, in the same way. The framework for the policy analyses, the guide for the interviews, the questionnaire, and the detailed design for the survey were consistent in all countries. Country teams were responsible for data collection in their own country and worked in close collaboration with the respective WP leaders who were overseeing the exercise in each country, particularly to ensure fidelity to the agreed procedures. Data analyses were led by WP leaders.

**FIGURE 2:**
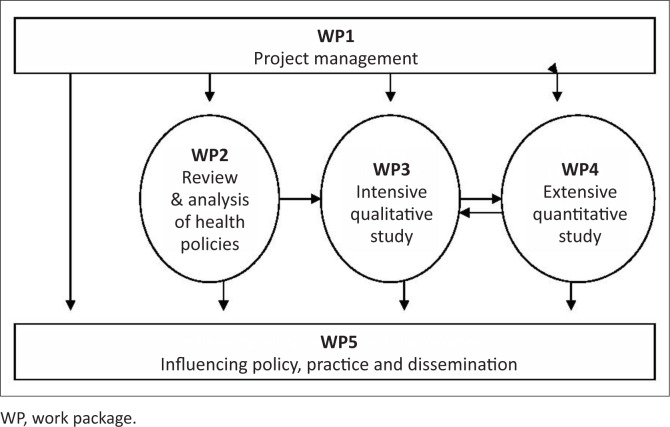
Schematic representation of the relationship between the five work packages in EquitAble

Figure 2 illustrates how we operationalised the management of the research programme into distinct WPs. The rationale, operational details and leadership for each of the WPs were discussed in detail and unanimously agreed upon. WPs 1 and 5 were led by Trinity College Dublin, WP2 by Ahfad University, WP3 by Stellenbosch University, and WP4 by SINTEF.

The management structure provided a sound basis for shared responsibility and participation by assigning responsibility for the different WPs to four different partners. All WPs were organised under one lead person and institution with counterparts in each of the four countries where the research was carried out. The lead person in each of these countries joined the respective WP teams and led the country team for the specific WP. Whilst intensive dialogue, including electronic, telephonic and face-to-face meetings aimed at establishing a ground for influence on the design in each WP, took place, the country teams also had sufficient flexibility to adapt to their own context and circumstances. The cost of such a flexible involvement approach may have been that some differences in research design and data collection were evident between the four project countries. However, the gain was assumed to be ownership, relevance and increased possibilities for utilisation of research results.

### Capacity building

Capacity building within the project took place on different levels. Firstly, country teams comprised both experienced senior researchers who led the project in their respective countries, and more junior researchers who acted as research assistants whilst working towards a Masters or PhD. Their study research was supervised by the senior researchers with whom they were working. Secondly, junior staff were involved in the publications of the different WPs, including the drafting of manuscripts, and inclusion in the authorship of publications. Thirdly, as mentioned previously, the WP leads were selected because of their particular experience and capacity, providing supervision to the country teams throughout the research process. Finally, the four large annual meetings that took place during the research process, each in a different country, involved as many different country team members as possible, regardless of their seniority. This provided an excellent opportunity for mutual learning and exchange amongst the participants, including North-South, South-North, South-South and North-North. The research teams that included people with disabilities also afforded mutual learning from each other’s perspectives and experiences. This included transfer of cultural and context-specific knowledge, providing experience of great importance for both the overall design and methods. The coordinator, project manager and WP leads all put substantial effort into creating working relationships and an atmosphere of ‘valuing-all-perspectives’, which influenced the research process, the design, and the utilisation of results.

The publication protocols and rules for joint publications, both of the overall project and in the country teams, were discussed extensively and were agreed upon by the team members. The publication protocol was first drafted at a meeting in Sudan, with reference to the Sudanese-led WP, and was subsequently adopted for all publications of the project in a meeting in Norway.

The project was officially launched in Sudan in March 2009, following a year of financial and administrative negotiations with the European Commission. In addition to its commencement in Sudan, the consortium also held its annual and closing meetings in Sudan, and also held annual meetings in Norway, South Africa, and Zimbabwe. Members of an external advisory board attended some of these meetings with participation by members from all of the countries and the institutional partners.

## Method

On completion of the research, each of the 23 people who had participated in the consortium, including administrative, research, academic, and civil society staff, were emailed inviting them to complete anonymously a bespoke questionnaire designed by the coordinating team (M.M., H.M., and J.M.V.). The 30-item questionnaire was divided into six thematic sections: content issues (1), context issues (2), process issues (3), impact (4), future (5), and comments (6). The administered questionnaire is provided in Appendix 1. Anonymity was protected by asking participants in the survey to email their responses to a colleague at Trinity College Dublin who was not associated with the project. This person printed off the response sheets and gave them to the coordinating team without indicating which response belonged to whom. The maximum response time allowed was 5 days.

## Results

Ten women and ten men responded to the questionnaire. Responses were from all countries and partners but were anonymous and could thus not be disaggregated in terms of countries or representative organisations. Quantitative scores from the 20 respondents were tabulated, indicating scores to each quantitative question within each thematic group. All scores reported below were rated on a 5-point Likert scale: strongly disagree (1), disagree (2), neither agree nor disagree (3), agree (4), and strongly agree (5).

### Content

Mean and modal scores indicate a high level of satisfaction, although the range of scores regarding the number of good quality publications included a rating of 1 by one respondent (Table 1). Generally, respondents were satisfied that the project addressed its stated target and delivered on all contractual obligations (comparative analysis reports related to policy and summary analysis of qualitative and quantitative data) related to all WPs, produced good peer-reviewed journal publications, and was relevant to people with disabilities. On content, the prevalent sentiment is captured by this comment provided by a project member who responded to the administered survey, ‘content issues of EquitAble were very pertinent to the continent [*Africa*] and to the priorities worldwide’^1^. Another respondent indicated that content not only focused on the ‘challenges facing people with disabilities, but also include[*d*] the broader issue of social inclusion for other marginalised or vulnerable groups’. Strength of the content was signified by another respondent who emphasised that the project provided scientific evidence to support already-existing anecdotal evidence in the participating countries, in the comment ‘documented the evidence base of what was known more anecdotally in the countries participating’.

**TABLE 1:** EquitAble content issues.

Question no.	Questionnaire statement	Total	Min.	Max.	Mean	Median	Mode	s.d.
**1**	I feel that EquitAble has addressed the issues which were outlined in the project documents.	20	4	5	4.65	5	5	0.49
**2**	I feel that, to date, EquitAble has produced a good number of quality publications related to disability and access to healthcare.	20	1	5	4.00	4	4	0.97
**3**	I feel that the research undertaken in EquitAble is relevant to people with disabilities.	20	4	5	4.65	5	5	0.49

Min., minimum, Max., maximum, s.d., standard deviation.

Content areas that respondents considered partially fulfilled with room for improvement, included ‘the area of translation of the research evidence into practical feedback strategies’, and the ‘need to write papers that link the work packages of the study’. With regards to publications, many respondents felt that whilst publications from WP2, the policy WP, had been very satisfactory (see e.g. Amadhila *et al*. 2013; Amin *et al*. 2011; MacLachlan *et al*. 2012), there were too few arising from WP3, the qualitative WP (Braathen *et al*. 2013; Van Rooy *et al*. 2012). In relation to the publication protocol, one respondent indicated that its operationalisation was ‘unfair for researcher[*s*] at lower level [*early career*] in [*their*] country team’. Other issues on publication related to a lack of time to analyse data from WP4, the quantitative WP, within the funding period. It was suggested that separate funding be secured to promote publication from the latter WP in particular, and also for dissemination and training on use of the policy analysis framework, ‘EquiFrame’, developed as part of WP2.

### Context

Respondents indicated broad satisfaction concerning contextual sensitivity to socio-economic and cultural differences between and within countries, and that a multi-country collaboration was a satisfactory way to undertake the project (Table 2). Research was seen as contextually relevant and commended that the project did ‘not impose outside researchers on countries.’ This was seen as a key factor in upholding contextual sensitivity. The following response sums up this value-led approach:

‘A key feature of EquitAble research relates to the sourcing of locally available research resource persons, especially field research assistants (including persons with disabilities), and individualised training workshops to match the diverse talent pool (college graduates; diploma holders; school drop-outs) in each one of the project sites within the four countries.’

**TABLE 2:** EquitAble context issues.

Question no.	Questionnaire statement	Total	Min.	Max.	Mean	Median	Mode	s.d.
**5**	I feel that EquitAble has been sensitive to the realities of conducting research in the socio-economic contexts of the African countries it has worked in.	20	3	5	4.35	4	4	0.59
**6**	I feel that EquitAble has been sensitive to the realities of conducting research in the cultural contexts of the African countries it has worked in.	20	4	5	4.35	4	4	0.49
**7**	I feel that working in a multi-country (and South-North) consortium has been an effective mechanism for addressing the project’s research questions.	20	3	5	4.50	5	5	0.69

Min., minimum, Max., maximum, s.d., standard deviation.

Another widespread view on the nature and impact of the collaboration was summed up by one respondent who stated:

‘The EquitAble project is an excellent example of how the North and South should work. The process involved all stakeholders from development of the proposal to implementation and publications. In all countries ethical approval was obtained from relevant IRBs [*Institutional Review Boards*].’

Collaboration amongst and between researchers from this South-North partnership was not without its complexities. The following statement provides a snapshot of some of the difficulties related to mutual respect and working relationships:

‘I think the critical issue here is how to handle/tackle issues that undermines each other’s dignity. Mixing North and South researchers offers us a window of opportunity to deal with some of these issue[*s*]/shortcomings of not handling each other appropriately.’

### Process

In line with the focus of our interest, we asked relatively more questions about the process of working together than any other theme. By and large, respondents felt that their voices had been heard when they made a contribution, and that the perspective of persons with disabilities and their representative organisations was sought (Table 3). Recognising the complexities of South-North partnerships, relationships between country-team members, across country teams, and with the project coordinators and WP leaders, all scored highly. Initial challenges of coordinating this multi-country and multidisciplinary study were overcome, as one respondent noted:

‘Perception is that initially it was difficult to coordinate different voices that were relating to different needs and contextual issues – especially via e-mail without face-to-face discussions – this improved when the project team met as a whole group.’

**TABLE 3:** EquitAble process issues.

Question no.	Questionnaire statement	Total	Min.	Max.	Mean	Median	Mode	s.d.
**9**	I feel that my voice has been heard when I have contributed to project meetings or email discussions.	20	3	5	4.40	5	5	0.75
**10**	I feel that the perspective of people with disabilities has been recognised within the overall project.	20	2	5	4.15	4	4	0.81
**11**	I feel that the relationship between myself and my country-team members has been good.	19	3	5	4.79	5	5	0.54
**12**	I feel that the relationship between my own country team and other country teams has been good.	20	3	5	4.25	4	4	0.72
**13**	I feel that the relationship between my own country team and the coordination team (in Ireland) has been good.	20	3	5	4.35	5	5	0.81
**14**	I feel that the relationship between my own country team and work package leaders has been good.	20	4	5	4.40	4	4	0.50
**15**	I feel that the relationship between my own country team and the FP7 office in Brussels has been good.	19	1	4	3.32	3	4	1.00

Min., minimum, Max., maximum, s.d., standard deviation; FP7, European Commission Seventh Framework Programme.

The publication protocol was highlighted in terms of an enabling process with one respondent indicating that:

‘Establishing the publication protocol through consensus was a real strength of the process. Also, country teams having direct access to both work package leaders and project coordinator[*s*] meant real time field challenges were addressed at once.’

Respondents were however least satisfied with their experience in relation to research administration of the project, particularly liaising with the European Commission’s Seventh Framework Programme (FP7) Office. In this regard one respondent stated:

‘I think this has been a happy and productive project, with lots more yet to come from it. I don’t feel that the EC requirements are necessarily overly burdensome – I think we should be very accountable for the large sum of money entrusted to us – however, the continual moving of the ‘goalposts’ in terms of what is required by Brussels, is really problematic and frustrating for all, including, I am sure, Commission staff in Brussels.’

Two distinct research administration challenges were highlighted. They were: ‘The constant change of project officer in Brussels is a challenge to maintain communications’, and ‘the constant change without automatic notification of the FP7 Participants Portal (on-line reporting mechanisms)’.

### Impact

Respondents expressed greater satisfaction with publications than influencing behavioural changes in practitioners, influencing policy development or revision, or heightening the profile of disability for African governments (Table 4). Respondents however recognised the initiative that each team took in influencing policy development or revision. In particular in Malawi the research team facilitated a policy workshop which resulted in developing Malawi’s first National Health Policy, based on EquitAble policy analyses findings.

**TABLE 4:** EquitAble impact.

Question no.	Questionnaire statement	Total	Min.	Max.	Mean	Median	Mode	s.d.
**17**	I feel that, to date, EquitAble has had a good impact in terms of academic publications.	20	2	5	4.05	4	4	0.76
**18**	I feel that EquitAble has had a good impact in terms of practitioners who are aware of the project changing their behaviour to be more inclusive of people with disabilities.	20	2	5	3.45	3	3	0.76
**19**	I feel that, to date, EquitAble has had a good impact in terms of influencing policy development or revision in at least some countries.	20	2	5	3.85	4	4	0.81
**20**	I feel that EquitAble has contributed to heightening the profile of access to health for people with disability, among African governments.	20	3	5	3.95	4	4	0.69

Min., minimum, Max., maximum, s.d., standard deviation.

### Future

Respondents felt that more time should be spent on publishing data, especially from WPs 3 and 4, but enthusiasm for giving time to influencing policy and practice was also strong (Table 5). No open questions were asked on this theme.

**TABLE 5:** EquitAble future.

Question no.	Questionnaire statement	Total	Min.	Max.	Mean	Median	Mode	s.d.
**21**	I feel that we need to invest more time in publishing the data from work package 2.	20	2	5	3.45	3	3	1.05
**22**	I feel that we need to spend more time on influencing policy and practice using the data from WP2.	20	2	5	4.15	4	5	0.93
**23**	I feel that we need to invest more time in publishing the data from work package 3.	20	3	5	4.20	4	4	0.77
**24**	I feel that we need to spend more time on influencing policy and practice using the data from WP3.	20	3	5	4.35	4	4	0.67
**25**	I feel that we need to invest more time in publishing the data from work package 4.	20	3	5	4.70	5	5	0.57
**26**	I feel that we need to spend more time on influencing policy and practice using the data from WP4.	20	3	5	4.50	5	5	0.69

WP, work package, Min., minimum, Max., maximum, s.d., standard deviation.

### Other comments

On working together with the same consortium in the future, the vast majority gave a resounding ‘Yes’ (19 out of 20). In relation to the following question, ‘What was the best thing about working in EquitAble?’, several participants mentioned as younger researchers the opportunity to complete a thesis as part of the EquitAble research project: but it was indicated that more experienced researchers also benefited. Several respondents returned to process issues as the ‘best thing’, for instance: ‘The implementation of the project has been carried out in a participatory manner with all project partners being involved.’ Finally, many respondents referred to being part of a multi- or interdisciplinary team, and several respondents referred to the enjoyment and benefit of the annual face-to-face meetings, which usually consisted of 20–30 participants across 2–3 days, each in beautiful and stimulating locations across different countries. In relation to the question, ‘what has been the worst thing about being involved in EquitAble?’, several respondents mentioned financial, administrative or reporting issues. Some were concerned with missed opportunities with regards to using much of the collected data, whilst others noted the challenges of broad participation: ‘Time consumed in reaching [*con*]census in different issues.’ See Box 1 for further comments offered by respondents.

BOX 1 **EquitAble Questionnaire comments [*sic*].**

‘A four-year multi-county; multi-disciplinary; multi-million research project which started and concluded with the “same set of researchers” is a testament to the consortium partners who were willing to share, learn, and grow with healthy respect to diversity of opinions and skill sets.’

‘The networking for me as young African researcher was valuable whereby being part of a multi research approaches and having access to reputable publications was a great experience On a national level it I became more involved with PWD [*persons with disabilities*] federations in similar reaches and training programs.’

‘The inevitable cultural, social and communication challenges of being part of such a large and culturally diverse consortium. This has been both the worst and best thing about EquitAble. On the one hand the diversity of the consortium members has led to a stronger project design; more culturally and contextually relevant. On the other hand I feel that it weakened some of the methodological procedures, particularly in WP2 and WP3, which were more open to individual interpretation than WP4.’

‘We did not always agree – the disagreements themselves were more related to systemic and contextual issues more than personal issues and we can only learn from this.’

‘The opportunity to work a multi-disciplinary research team with differing levels of research experience in creation of empirical evidence to further universal access to healthcare was insightful. The opportunity to build research capacity in an evolving area of research was intellectually invigorating. The ethos of learn, share, and grow made it possible for early career researchers and advanced career researchers to prosper.’

‘How the project is organized that sometimes those at the base felt left out in critical decision making with regard to the project.’ ‘Having to access houses and seeing the suffering of disabled people and being unable to offer any assistance and only fill very long questionnaire was very hard. Until now I still have contacts with some of the families where you try to assist. Equitable was very emotional research project and that is sometime get missed from scientific publications.’

WP, work package.

## Discussion

This study explored the content, context, process and impact of the research undertaken, and asked for general comments and ideas for the future. Overall, it is clear that the 20 respondents felt that their participation in the project as members of the project team was a positive experience. Most participants would be keen to work together again. Of particular note was that respondents felt that the project successfully addressed its stated content targets, was conducted in a way appropriate to different cultural and socio-economic contexts, and engendered a process of participation and mutual learning.

Some specific issues are worth highlighting. Given that large projects often end their funding period when much analyses, publication and dissemination remains to be carried out, one challenge will be to maintain motivation and coherence of activity between team members who may be working on new projects.

EquiFrame has already been used to write new, and revise existing, health policies in Sudan, Malawi and South Africa. We hope country teams will continue to monitor the impact of this framework on policy revision and development, and contribute to monitoring and evaluation of the real impact of policies. The impact of EquiFrame (Mannan *et al*. 2014) already reaches beyond Africa, with Handicap International translating it into French for use by civil society organisations internationally, and the United Nations Educational, Scientific and Cultural Organisation (UNESCO) organising a conference on its potential use in contributing to their social inclusion work in South-East Asia. Findings from EquiFrame have also been presented at leading regional and international fora such as the African Union Social Affairs ministerial summit in Khartoum (Dube *et al*. 2010), and the United Nations Commission for Social Development in New York (MacLachlan 2012).

Returning to our own capacity building within the research team, several participants undertook MSc or PhD degrees as part of the project and have indicated benefits from being part of a multi-country and multidisciplinary team. The project has thus presented students with the opportunity to participate in a large complex project and some of these students may go on to lead such projects in the future. Whilst most participants felt that the research protocol was a strength of the project, it having been agreed in open discussion in project meetings (Appendix 2), some suggested that this might be unfair to early career researchers. In particular, few of the early career researchers had experience of publishing prior to involvement in project EquitAble. Despite this, Namibia and South Africa facilitated early career researchers to publish as lead authors on multi-authored papers (Amadhila *et al*. 2013; Braathen *et al*. 2013), and it has been made clear that any individual can be the lead of a publication if they initiate it.

Indeed, one of the team’s greatest challenges is to do justice to the enormous amount of data collected in WP4, the quantitative WP, as yet unpublished in peer-reviewed journals. Whilst as per the contractual obligations to the European Commission, a summary of the analyses has been submitted, the team has a moral responsibility to ‘make public’ (peer-reviewed submissions) data that has been provided to the team by thousands of participants. Further funding support should be sought to facilitate detailed analyses for publishing in peer-review journals.

The EquitAble research project has achieved many of its objectives in terms of enabling vulnerable individuals’ voices and those of researchers themselves to be heard. The inclusion of representatives of the SADPD in the consortium and in the project team provided a good grounding for dialogue between researchers and civil society. Individuals’ voices from different communities and backgrounds were heard and communicated through the research team throughout data collection and dissemination. Although the project had a full year of research analyses and the writing up of this data following data collection comprised in the schedule, it was still not enough, and much of the data from the project still awaits analyses and publication. More time for analyses and write up should be funded and more members of the team should be encouraged to lead the write up of data for dissemination. Whilst writing workshops can empower less experienced researchers, the nature of their own contracts often means that they have insufficient time to give to the writing up of research, although many would like to do this, recognising that it would be advantageous for their own careers. The development of ‘research-writing mentors’, including outside the research team, could be one way of addressing this issue.

The quest to gain understanding and solutions on issues of equitable access to health services for vulnerable groups was the core aim that brought together the EquitAble project partners. It is this quest that kept the group bonded together and ensured sustainability of the project despite our differences.

## Conclusion

The EquitAble project was a multi-country and multidisciplinary project, which sought to identity factors influencing access to health care for vulnerable groups in four African countries. The project was seen as an enjoyable and appropriate process by the team who have an acute awareness that they have responsibilities to continue analysing, publishing and disseminating results, and who look forward to working together in the future.

The authors are aware that in commenting on our own team processes in this article, we are both the image and the reflection. Others may view our work differently. This article has attempted to be reflective in a structured way: a less structured approach may have highlighted different themes and allowed greater scope for individuals to express their distinct views. We hope that other perspectives may yet be forthcoming. If, in conducting social research, we are ‘to be the change we seek’, then this requires a variety of reflective methodologies, none of which can be expected to offer a complete image or to position itself in an impartial or neutral space. Nonetheless, we hope that the willingness of our own consortium to engage in this reflexive analysis will encourage other research teams to undertake similar ‘learning from doing’ assessments, and by doing this, help to identify good practices that would help research partnerships to achieve their aims.

Whilst recognising that a broad range of learning about project context, process and impact can be drawn from this particular project, we conclude by synthesising and highlighting 10 primary points of recommendation that have been noted by members of our consortium (further recommendations for future research nor capacity building are provided in Appendix 3):

- Engender and nurture a consciousness around establishing joint ownership and participation amongst all partners of a complex research project, and allow them to see how they are interdependent.
- Ensure to have proposal development meetings with all potential partners in attendance so that they feel that they are part of it from the start.
- Enable leadership opportunities for all participating consortium partner institutions so that each can lead on some aspects of the project.
- Establish agreement on a publication protocol and an effective implementation mechanism through clear and open discussion, and publish the protocol on the project website.
- Include disabled people’s organisations and related civil society organisations as active members of the research team, each with their own dedicated funding.
- Provide opportunities to develop consortium partners’ research and research administration skills to enable them to meet European Commission or other funding agency requirements.
- Accept that even with clear and unanimously agreed protocols, not everyone will abide by them and there is little to be gained by engendering conflict over these instances.
- Promote dissemination and influencing opportunities by including research-users at an early stage of the research: we used ‘consultation workshops’, gaining much insight and authenticating our consultative processes.
- Build funding for research-writing workshops and research-writing mentors into research proposals, targeted especially at less experienced researchers.
- Be well-prepared for project meetings, anticipate and try to address difficulties in private, be diplomatic in public and strive to retain the trust and respect of team members for each other.

## Footnotes

1 All participant responses to the survey were provided anonymously. Participants’ comments are therefore outlined throughout this article within quotation marks without demographic information of respondents for individual comments.

## Appendix 1 Reflections on the experience of working as part of the EquitAble Consortium

This questionnaire seeks to capture some of your views about and experience of working as a Partner in EquitAble. Respondents are assured that the information that they provide will be treated as strictly confidential. Your anonymity will be protected by asking you to e-mail your responses to our colleague (name provided) (who has not been associated with EquitAble) and who will print off your response sheets and give them to us, and then delete your e-mail, without indicating which response belongs to whom.

Please therefore send your completed responses to (e-mail address provided) by (date provided).

Please indicate your responses on the scales provided by placing an “X” in the box that corresponds with your views.

Please complete the blank windows with typed responses to indicate your ‘open’ comment responses.

Please indicate, with regard to the following statements, if you:

**Table:**

1.2.3.4.5.	Strongly disagree
	Disagree
	Neither Agree or Disagree
	Agree
	Strongly Agree

### 1 Equitable content issues

1 I feel that EquitAble has addressed the issues which were outlined in the project documents.
  **Table:**
  *Strongly Disagree*	*Disagree*	*Neither Agree or Agree*	*Agree*	*Strongly Agree*
  1	2	3	4	5
2 I feel that, to date, EquitAble has produced a good number of quality publications related to disability and access to healthcare.
  **Table:**
  *Strongly Disagree*	*Disagree*	*Neither Agree or Agree*	*Agree*	*Strongly Agree*
  1	2	3	4	5
3 I feel that the research undertaken in EquitAble is relevant to people with disabilities.
  **Table:**
  *Strongly Disagree*	*Disagree*	*Neither Agree or Agree*	*Agree*	*Strongly Agree*
  1	2	3	4	5
4 Any Comments on other EquitAble Content issues?

### 2 Equitable Context Issues

5 I feel that EquitAble has been sensitive to the realities of conducting research in the socio-economic contexts of the African countries it has worked in.
  **Table:**
  *Strongly Disagree*	*Disagree*	*Neither Agree or Agree*	*Agree*	*Strongly Agree*
  1	2	3	4	5
6 I feel that EquitAble has been sensitive to the realities of conducting research in the cultural contexts of the African countries it has worked in.
  **Table:**
  *Strongly Disagree*	*Disagree*	*Neither Agree or Agree*	*Agree*	*Strongly Agree*
  1	2	3	4	5
7 I feel that working in a multi-country (and South-North) consortium has been an effective mechanism for addressing the project’s research questions.
  **Table:**
  *Strongly Disagree*	*Disagree*	*Neither Agree or Agree*	*Agree*	*Strongly Agree*
  1	2	3	4	5
8 Any Comments on EquitAble Context Issues?

### 3 Equitable process issues

9 I feel that my voice has been heard when I have contributed to project meetings or e-mail discussions.
  **Table:**
  *Strongly Disagree*	*Disagree*	*Neither Agree or Agree*	*Agree*	*Strongly Agree*
  1	2	3	4	5
10 I feel that the perspective of people with disabilities has been recognised within the overall project.
  **Table:**
  *Strongly Disagree*	*Disagree*	*Neither Agree or Agree*	*Agree*	*Strongly Agree*
  1	2	3	4	5
11 I feel that the relationship between myself and my country-team members has been good.
  **Table:**
  *Strongly Disagree*	*Disagree*	*Neither Agree or Agree*	*Agree*	*Strongly Agree*
  1	2	3	4	5
12 I feel that the relationship between my own country team and other country teams has been good.
  **Table:**
  *Strongly Disagree*	*Disagree*	*Neither Agree or Agree*	*Agree*	*Strongly Agree*
  1	2	3	4	5
13 I feel that the relationship between my own country team and the Coordination Team (in Ireland) has been good.
  **Table:**
  *Strongly Disagree*	*Disagree*	*Neither Agree or Agree*	*Agree*	*Strongly Agree*
  1	2	3	4	5
14 I feel that the relationship between my own country team and Work Package Leaders has been good.
  **Table:**
  *Strongly Disagree*	*Disagree*	*Neither Agree or Agree*	*Agree*	*Strongly Agree*
  1	2	3	4	5
15 I feel that the relationship between my own country team and the FP7 Office in Brussels has been good.
  **Table:**
  *Strongly Disagree*	*Disagree*	*Neither Agree or Agree*	*Agree*	*Strongly Agree*
  1	2	3	4	5
16 Any comments on other EquitAble Process Issues?

### 4 Equitable impact

17 I feel that, to date, EquitAble has had a good impact in terms of academic publications.
  **Table:**
  *Strongly Disagree*	*Disagree*	*Neither Agree or Agree*	*Agree*	*Strongly Agree*
  1	2	3	4	5
18 I feel that EquitAble has had a good impact in terms of practitioners who are aware of the project changing their behaviour to be more inclusive of people with disabilities.
  **Table:**
  *Strongly Disagree*	*Disagree*	*Neither Agree or Agree*	*Agree*	*Strongly Agree*
  1	2	3	4	5
19 I feel that, to date, EquitAble has had a good impact in terms of influencing policy development or revision in at least some countries.
  **Table:**
  *Strongly Disagree*	*Disagree*	*Neither Agree or Agree*	*Agree*	*Strongly Agree*
  1	2	3	4	5
20 I feel that EquitAble has contributed to heightening the profile of access to health for people with disability, among African governments.
  **Table:**
  *Strongly Disagree*	*Disagree*	*Neither Agree or Agree*	*Agree*	*Strongly Agree*
  1	2	3	4	5

### 5 Equitable future

21 I feel that we need to invest more time in publishing the data from Work Package 2.
  **Table:**
  *Strongly Disagree*	*Disagree*	*Neither Agree or Agree*	*Agree*	*Strongly Agree*
  1	2	3	4	5
22 I feel that we need to spend more time on influencing policy and practice using the data from WP2.
  **Table:**
  *Strongly Disagree*	*Disagree*	*Neither Agree or Agree*	*Agree*	*Strongly Agree*
  1	2	3	4	5
23 I feel that we need to invest more time in publishing the data from Work Package 3.
  **Table:**
  *Strongly Disagree*	*Disagree*	*Neither Agree or Agree*	*Agree*	*Strongly Agree*
  1	2	3	4	5
24 I feel that we need to spend more time on influencing policy and practice using the data from WP3.
  **Table:**
  *Strongly Disagree*	*Disagree*	*Neither Agree or Agree*	*Agree*	*Strongly Agree*
  1	2	3	4	5
25 I feel that we need to invest more time in publishing the data from Work Package 4.
  **Table:**
  *Strongly Disagree*	*Disagree*	*Neither Agree or Agree*	*Agree*	*Strongly Agree*
  1	2	3	4	5
26 I feel that we need to spend more time on influencing policy and practice using the data from WP4.
  **Table:**
  *Strongly Disagree*	*Disagree*	*Neither Agree or Agree*	*Agree*	*Strongly Agree*
  1	2	3	4	5

### 6 Equitable comments

27 Would you be willing to collaborate in another consortium with the same Partners as in EquitAble?
  **Table:**
  YES	NO
  Please provide a reason(s) for your answer in the space provided below:
28 What has been the best thing about being involved in EquitAble?
29 What has been the worst thing about being involved in EquitAble?
  **Table:**
  *Strongly Disagree*	*Disagree*	*Neither Agree or Agree*	*Agree*	*Strongly Agree*
  1	2	3	4	5
30 What other comments would you like to make about EquitAble?

## Appendix 2 EquitAble Project publication protocol

Published papers:

- Work package (WP) leader decides* authorship for comparative paper(s) in consultation with the project coordinator.
- Country coordinators decide* authorship for country papers in consultation with the project coordinator.
- Authorship should be guided by criteria set out by the International Committee of Medical Journal Editors (http://www.ICMJE.org).

Conference papers:

- Oral or poster presentations should be approved* by the WP and country leaders concerned, in consultation with the project coordinator.

## Appendix 3 EquitAble: Recommendations for future research and capacity building

- Planned and budgeted data analyses and writing workshops hosted by work package (WP) leaders.
- Seeking FP7 national contact points to seek participation of persons with disabilities and their representative organisations in drawing up research priorities.
- Institutional research administration capacity building through national contact points at country level.
- Identification of publication opportunities for early career researchers based on country specific data.

## References

[CIT0001] Amadhila E., Van Rooy G., McVeigh J., Mannan H., MacLachlan M. & Amin M., 2013, ‘Equity and core concepts of human rights in Namibian health policies’, *Africa Policy Journal* 8, 34–45.

[CIT0002] Amin M., MacLachlan M., Mannan H., El Tayeb S., El Khatim A., Swartz L. et al., 2011, ‘EquiFrame: A framework for analysis of the inclusion of human rights and vulnerable groups in health policies’, *Health and Human Rights* 13(2), 82–101. 22957368

[CIT0003] Braathen S.H., Vergunst R., Mji G., Mannan H. & Swartz L., 2013, ‘Understanding the local context for the application of global mental health: A rural South African experience’, *International Health* 5(1), 38–42. http://dx.doi.org/10.1093/inthealth/ihs016 24029844 10.1093/inthealth/ihs016

[CIT0004] Brannen J., 2005, ‘Mixing methods: The entry of qualitative and quantitative approaches into the research process’, *International Journal of Social Research Methodology* 8, 173–184. http://dx.doi.org/10.1080/13645570500154642

[CIT0005] Dube A.K., MacLachlan M., Amin M. & Mannan H., 2010, ‘Equitable access to healthcare and persons with disabilities’, presentation at Khartoum, African Union Social Affairs Ministerial Summit, 21–23 November.

[CIT0006] MacLachlan M., 2012, ‘Community based rehabilitation and inclusive global health: A way forward’ statement to the United Nations Commission for Social Development, New York, 02 February.

[CIT0007] MacLachlan M., Amin M., Mannan H., El Tayeb S., Bedri N., Swartz L. et al., 2012, ‘Inclusion and human rights in health policies: Comparative and benchmarking analysis of 51 policies from Malawi, Sudan, South Africa and Namibia’, *PLoS ONE* 7(5), e35864 http://dx.doi.org/10.1371/journal.pone.0035864 22649488 10.1371/journal.pone.0035864 PMC3359320

[CIT0008] Mannan H., Amin M., Maclachlan M. & EquitAble consortium, 2014, *The EquiFrame manual: An analytical tool for evaluating and facilitating the inclusion of core concepts of human rights and vulnerable groups in policy documents*, 2nd edn., Global Health Press, Dublin.

[CIT0009] Van Rooy G., Amadhila E.M., Mufune P., Swartz L., Mannan H. & MacLachlan M., 2012, ‘Perceived barriers to accessing health services among people with disabilities in rural Northern Namibia’, *Disability & Society* 27(6), 761–75. http://dx.doi.org/10.1080/09687599.2012.686877

